# Supplementation with Citrus Low-Methoxy Pectin Reduces Levels of Inflammation and Anxiety in Healthy Volunteers: A Pilot Controlled Dietary Intervention Study

**DOI:** 10.3390/nu16193326

**Published:** 2024-09-30

**Authors:** Amrita Vijay, Anthony Kelly, Suzanne Miller, Melanie Marshall, Althea Alonso, Afroditi Kouraki, Catherine Probert, Elizabeth J. Simpson, Ana M. Valdes

**Affiliations:** 1 NIHR Nottingham Biomedical Research Centre and Academic Unit of Injury, Recovery and Inflammation Science, School of Medicine, University of Nottingham, Nottingham NG7 2RD, UK; amrita.vijay@nottingham.ac.uk (A.V.); tony.kelly@nottingham.ac.uk (A.K.); suzanne.miller@nottingham.ac.uk (S.M.); afroditi.kouraki1@nottingham.ac.uk (A.K.); 2 School of Life Sciences, University of Nottingham, Nottingham NG7 2RD, UK; melanie.marshall@nottingham.ac.uk (M.M.); liz.simpson@nottingham.ac.uk (E.J.S.); 3 School of Agriculture and Food Sciences, University College Dublin, D04 C1P1 Dublin, Ireland; althea.alonso@ucdconnect.ie; 4 Translational Medical Sciences, School of Medicine, Biodiscovery Institute, University of Nottingham, Nottingham NG7 2RD, UK; catherine.probert@nottingham.ac.uk

**Keywords:** low-methoxyl pectin, dietary intervention, inflammation, anxiety

## Abstract

**Background/Objective:** Although low-methoxy (LM) pectin (polysaccharides extracted from citrus peels) can reduce inflammation by binding to and inhibiting the TLR-2 pathway in animal models and in vitro studies, the anti-inflammatory effects of LM pectin in humans and mood have not been explored to date. The purpose of this study is to assess the role of dietary supplementation with LM pectin in healthy volunteers on inflammatory markers and on mood, specifically anxiety and depression. **Methods:** We carried out a 4-week dietary intervention with LM citrus pectin on healthy volunteers (N = 14, age 40 ± 16 y, BMI 24.7 ± 3.0 kg/m^2^, sex F 57%) comparing the effects of daily supplementation with 20 g of LM citrus pectin versus 10 g of maltodextrin as the control (N = 15 age 43.2 ± 11 y, BMI 25.18 ± 2.0 kg/m^2^, sex F 66%). The effects on mood and inflammation were also tested with LM pectin at 5 g, 10 g and 15 g (2 weeks each) in an independent cohort of n = 15 healthy volunteers (age 36 ± 21 y, BMI 23.5 ± 2.4 kg/m^2^, sex F 80%). We assessed serum levels of TNF-alpha (downstream from TLR-2 activation), IL-1 beta, IL-6, IL-10, INF-gamma, CRP, zonulin and TLR-2 concentration which were measured using ELISA in blood samples collected at both the baseline and follow-up visits. Validated measures of anxiety and depression were collected at baseline and follow-up. **Results:** Supplementation with 20 g of LM pectin resulted in decreases in the pro-inflammatory markers TNF-alpha, IL-1 beta, IL-6 and INF-gamma (all *p* < 0.05) and an increase in anti-inflammatory marker IL-10 (*p* = 0.01) at the end of the 4 weeks. No such effects were observed in the control group. In addition, a significant drop in anxiety scores (from 8.38 to 4.46, *p* < 0.006) was found with the 20 g/day intervention but not in the control arm. In the dose–response study, anti-inflammatory effects were seen only at 15 g for TNFα (*p* < 0.003) and a suggestive increase in IL-10 (*p* = 0.08), alongside a drop in TLR-2 (*p* < 0.027). No significant anti-inflammatory effects were observed at 5 g and 10 g doses of LM pectin supplementation. Significant dose-dependent drops in both anxiety and depression scores were found with 10 g (*p* < 0.001) and 15 g per day (*p* < 0.0002). **Conclusions:** The current study identifies anxiety-reducing and anti-inflammatory effects of supplementation with 15 g/day LM pectin in healthy humans. Further research is needed to elucidate the precise mechanism and to validate the efficient dose and minimum duration of supplementation.

## 1. Introduction

Pectin is a naturally occurring polysaccharide present in all higher plants. Its main chain is mostly formed of α-D-galacturonic acid units linked via 1→4-glycosidic bonds [1]. The physical and biochemical characteristics of pectins are determined by the degree of methyl-esterification (DM) at the carboxyl groups in the galacturonic acid units [2]. Pectins with DM ≥ 50% are referred to as high-methoxyl (HM) pectin and those with DM < 50% are referred to as low-methoxyl (LM) pectin [2]. Previous research studies have shown anti-inflammatory effects of LM pectin ingestion in animal models, but lower or no effects with HM pectin [3,4]. The mechanism underlying the anti-inflammatory effects of LM pectin has been explored using in vitro models and it has been shown that LM pectins act, with higher affinity than HM pectins, as antagonists of Toll-like receptor 2 (TLR-2), particularly TLR-2/TLR-1 heterodimers [5]. Differential effects by LM vs. HM pectins on TLR-2 have also been validated in in vivo models [5]. TNF-alpha is a pro-inflammatory cytokine downstream of the activation of TLR-2 [6]; therefore, we hypothesise that levels of this cytokine should decrease in response to dietary supplementation with LM pectin.

In addition, since pectin is a type of dietary fibre, it can be fermented by gut microbes resulting in the production of short-chain fatty acids (SCFAs), mainly acetate followed by lower levels of propionate and butyrate, as demonstrated by in vitro gut models [7]. A higher production of SCFAs is linked to lower levels of inflammatory cytokines [8] and also lower levels of anxiety in animal models [9].

In general, systemic inflammation appears to be a factor contributing to anxiety [10,11]; therefore, any intervention that reduces systemic inflammation may, in principle, have the potential of relieving anxiety. More specifically, the anti-inflammatory effects of pectin have also been linked to decreases in anxiety and depression in animal models [12,13,14].

Post-stroke inflammatory responses are involved in increased depressive symptoms. Oral administration of apple and citrus pectin in a murine model of stroke (called ischemia/reperfusion (I/R) injury) resulted in a significant reduction in both inflammatory responses and depressive behaviours in animals [12]. Oral administration of apple and citrus pectins was found to yield a downregulation of proinflammatory cytokines in these animals and to have antidepressant-like effects on cerebral I/R-induced depression. The authors hypothesised that the effects observed could be explained by a reduction in inflammation in the brain hippocampus [12]. Similarly, the administration of tansy pectin resulted in decreased anxiety-related behaviour in mice [13], and in zebrafish, oral administration of citrus pectin reduced measures of anxiety via GABAergic neurotransmission [14].

The anti-inflammatory and potential anxiety and depression-relieving effects of ingesting LM pectin have not yet been studied in humans. We hypothesised that, if LM pectin indeed reduces low-grade systemic inflammation in healthy volunteers, this could translate into improvements in measures of mood given that chronic inflammation has been related to higher levels of anxiety and depression [15].

The objective of this study was therefore to assess the effect of dietary supplementation with LM pectin in healthy volunteers on inflammatory markers and on validated measures of anxiety and depression. The primary outcomes of such intervention were chosen to be changes in circulating levels of TNF-alpha (given its links to the TLR-2 pathway) and changes in anxiety and depression scores. Secondary outcomes were other pro-inflammatory (IL-1 beta, IL-6, IFN-gamma, CRP) and anti-inflammatory (IL10) cytokines. As exploratory markers, we also measured changes in circulating SCFAs and in the gut permeability marker zonulin.

For this, a pilot, randomised, parallel design, controlled four-week dietary intervention in 19 healthy volunteers was first carried out (NCT06580132), of which 15 were randomised to placebo (maltodextrin 10 g/day) and 14 to 20 g/d LM pectin administration. Subsequently, we recruited an additional 15 healthy volunteers and assessed the effects of escalating doses (from 5 to 15 g/d) of the same LM pectin in a single-group, longitudinal design.

## 2. Materials and Methods

### 2.1. Participants and Sample Collection

For both study cohorts, participants were recruited to the study at UoN from 10 November 2021 to 31 May 2024 (University of Nottingham Faculty of Medicine and Health Sciences Research Ethics Committee approval FMHS 302-0621 and FMHS 287-0523). All subjects provided written informed consent.

Participants were recruited from the community using posters, University internal mailings and via social media platforms, and were considered eligible if aged >18 years and had a body mass index (BMI) between 18.5 and 39.9 kg/m^2^. Participants were excluded if they had psychosocial or gastrointestinal conditions (e.g., malabsorptive conditions such as IBS/IBD, coeliac), were taking any regular medication (except contraception), were following or anticipated to commence a specialised commercially available weight loss diet and/or programme, were pregnant or breastfeeding, had a history or current psychiatric illness or a neurological condition (e.g., epilepsy) or had taken part in a research study in the last 3 months involving invasive procedures or an inconvenience allowance.

### 2.2. Study Cohort 1–20 g per Day vs. Placebo

**Figure 1:**
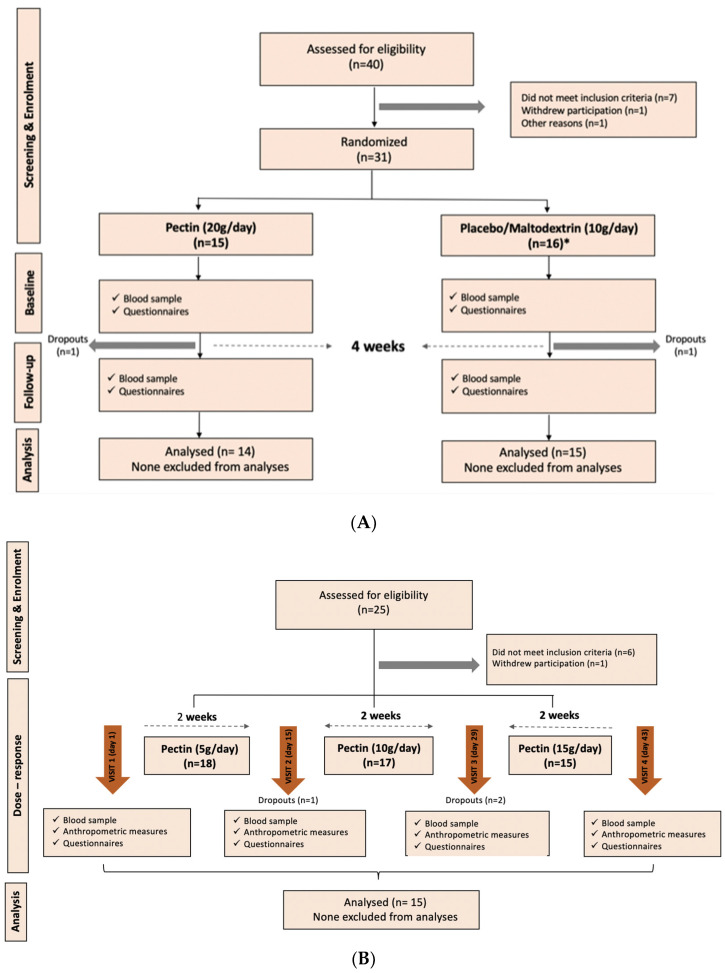
CONSORT diagrams of participant flow in the dietary intervention clinical study. (**A**) Flow of participants through each stage of the intervention study 1 (20 g per day vs. control) enrolment, allocation, follow-up and analysis, adhering to the CONSORT guidelines. (**B**) Flow of research participants for the dose–response cohort (intervention 2). * includes additional 10 controls from a separate 4-week dietary fibre intervention.

Eligible participants were randomised (using www.sealedenvelope.com) to either 20 g/d of LM citrus pectin with degree of methyl-esterification below 10% (DM < 10, supplied by CP Kelco, Lille Skensved, Denmark) per day or 10 g/d of maltodextrin (sourced from www.bulk.com) supplements for four weeks. They were given these supplements with standard scoops for measuring the doses. Participants were required to consume the powders daily for 4 weeks and were recommended to mix them into a morning drink, yoghurt or smoothie. They were advised to consume this as soon as possible due to their thickening properties when added to liquid. Samples and questionnaire data from an additional 10 control participants (10 g/day maltodextrin) who were part of a separate 4-week dietary fibre intervention study (FMHS 287-0523) were included in the current study cohort after matching for age, sex and BMI. The sample measures for n = 10 controls collected as part of a separate dietary intervention included the primary outcomes and all serum measures except for TLR-2, zonulin and short-chain fatty acids (see Figure 1A).

Fasting blood samples were collected from participants and anthropometric measures made prior to the intervention period (baseline; Visit 1) using standard procedures. They were also asked to complete the Hospital Anxiety and Depression Scale (HADS) questionnaire using an online platform. Based on the randomisation, participants were supplied with their respective pre-weighed supplement in food grade pouches.

Participants in both arms were asked to maintain a food diary in order to monitor adherence to the intervention. In addition, regular weekly contact was made via email by the study team to check that participants were not experiencing any issues and to encourage compliance. At the end of the 4 weeks, fasting blood samples were again collected, and questionnaire measures were again completed online by participants.

A total of 33 participants were enrolled, of which 29 completed the study, 14 in the pectin arm and 15 in the maltodextrin arm.

### 2.3. Study Cohort 2–Dose–Response Study

Participants were recruited using the same methods as previously described, with additional advertising via the social media platform of the “Nottingham Post”. This single-group, non-randomised dose–response study did not have a placebo arm. Participants were required to consume powdered, LM citrus pectin daily for 6 weeks and, as before, were recommended to mix this into a morning drink, yoghurt or smoothie. The initial dose of pectin consumed was 5 g/day and this was increased by 5 g every two weeks for 6 weeks (5 g for weeks 1 and 2, 10 g for weeks 3 and 4, escalating to 15 g for weeks 5 and 6).

Before the start of the intervention and at the end of each “dosing” period, fasting blood samples were collected from participants. Anthropometric measures were made, and participants were asked to complete a series of questionnaires. Participants were supplied with their pre-weighed supplement in food grade pouches and a dose scoop.

A total of 17 individuals were enrolled, of whom 15 completed the study. The CONSORT diagram of enrolment for both cohorts is shown in Figure 1.

### 2.4. Assessments

Sample collection: Venous blood was collected from participants using a BD vacutainer blood collection device (butterfly needle) into BD 5 mL yellow top Serum Separation Tubes. Samples were left to stand at room temperature for 15 min before being centrifuged for ten minutes at 3000 RCF. The resulting serum was aliquoted into 2 mL cryo-vials and stored immediately at −80 °C.

Inflammatory markers: Serum CRP, IL-6, TLR-2, TNF-alpha and zonulin (Haptoglobulin) levels were determined using ELISA. IL-6, TNF-alpha and Zonulin Quantkine ELISA kits were obtained from R&D systems, Minneapolis, MN, USA (HS600C, HSTA00E and DHAPG0, respectively). CRP concentration was measured in serum using R&D systems Quantikine Quick kit (QK1707), Minneapolis, MN, USA and TLR-2 measured using Invitrogen Human ELISA kit (EH459RB), ThermoFisher Scientific, Paisley, UK. Assays for IL-1beta, INF-gamma and IL-10 were performed by Affinity Biomarker Labs (London, UK) https://affinitybiomarkerlabs.com/, an ISO-15189 [16] accredited service company specialising in radioimmunoassays. The TLR-2 assays were performed two years later in the cohort 1 stored serum samples and at the same time as the cohort 2 samples were analysed. This was due to quantification issues faced with the initial test kits which were then resolved with new test kits that were ordered at a later stage and used to measure TLR-2 in the cohort 2 samples. All other inflammatory markers and the SCFAs were measured within 6 months from the time of the collection of serum samples.

Short-chain fatty acid (SCFA) measurements: Metabolomic profiling was performed on the serum samples from cohort 1 by Metabolon Inc., Morrisville, NC, USA using liquid chromatography coupled with tandem mass spectrometry (LC-MS/MS), as previously described [17]. In all the samples, the SCFAs acetate, propionate, butyrate, 2-methylbutyrate, isobutyrate, valerate and isovalerate, and the medium-chain fatty acid hexanoate were measured. For the sake of ease of reading, hexanoate is included in the definition of SCFAs.

Anxiety and Depression: The widely used 14-item Hospital Anxiety and Depression Scale HADS self-screening questionnaire includes subscales for depression and anxiety [18], which have a range of values from 0 to 21, with scores falling into four categories: normal (0–7), mild (8–10), moderate (11–14) and severe (15–21). The anxiety subscale includes questions that are related to feelings of tension, nervousness, worry and panic whilst the depression subscale includes questions that are focussed on feelings of low mood, lack of energy and enjoyment in daily activities and loss of interest in personal surroundings [18].

For gastrointestinal symptoms: For the first intervention, individuals were asked to list whether they had any gastrointestinal symptoms such as constipation, bloating, acid reflux or diarrhoea. For the second intervention, the Gastrointestinal Symptom Rating Scale (GSRS) was utilised. The GSRS is used to assess and measure the severity of gastrointestinal symptoms in individuals and has been widely validated [19]. It was developed to evaluate a wide range of symptoms related to the gastrointestinal tract, such as abdominal pain, bloating, diarrhoea, constipation and reflux [20]. The GSRS uses a seven-point Likert scale for each item, where 1 represents the absence of symptoms and 7 indicates very troublesome symptoms. Higher scores (closer to 7) indicate more severe and troublesome symptoms and are considered clinically significant. Scores towards the lower end of the scale (1 or 2) typically represent normal or mildly troublesome symptoms that might not require clinical intervention. In our study, the GSRS was used to enable participants to record tolerability, bloating and other adverse effects during the course of the intervention.

### 2.5. Statistical Analysis

In order to compare changes between baseline and follow-up, paired *t*-tests were used for all measures that did not violate assumptions of normality. To achieve normality, most biomarkers were log10-transformed. For measures whose distributions were not normal, non-parametric methods, specifically Wilcoxon matched-pairs signed rank tests, were carried out. This was the case only for anxiety and depression HADS scores. Analyses were performed using GraphPad Prism 10.2.0. *p*-values <0.05 were considered as statistically significant. In order to compare the effect of pectin vs. maltodextrin supplementation in the first intervention, we computed the change on any given measure between baseline and follow-up (Δ) for each individual for each measure. The distribution of Δpectin vs. Δmaltodextrin was compared using unpaired *t*-tests. *p* < 0.05 was considered as statistically significant.

## 3. Results

The descriptive characteristics of the two interventional cohorts, including the placebo group, are shown in Table 1. The age (40.3 vs. 43.2 years), sex (F = 57% vs. 66%) and BMI (24.5 vs. 25.2 kg/m^2^) were similar between both the pectin and placebo arms with none of the characteristics being significantly different. No adverse events were reported in any of the study groups. In the first interventional cohort, no significant differences in baseline demographics were seen between the control and 20 g groups (Table 1). In both the placebo and pectin groups, only one participant reported any GI symptoms at baseline, and two at follow-up. Neither the change from baseline to follow-up nor the prevalence between the two groups was statistically significant.

**Figure 2:**
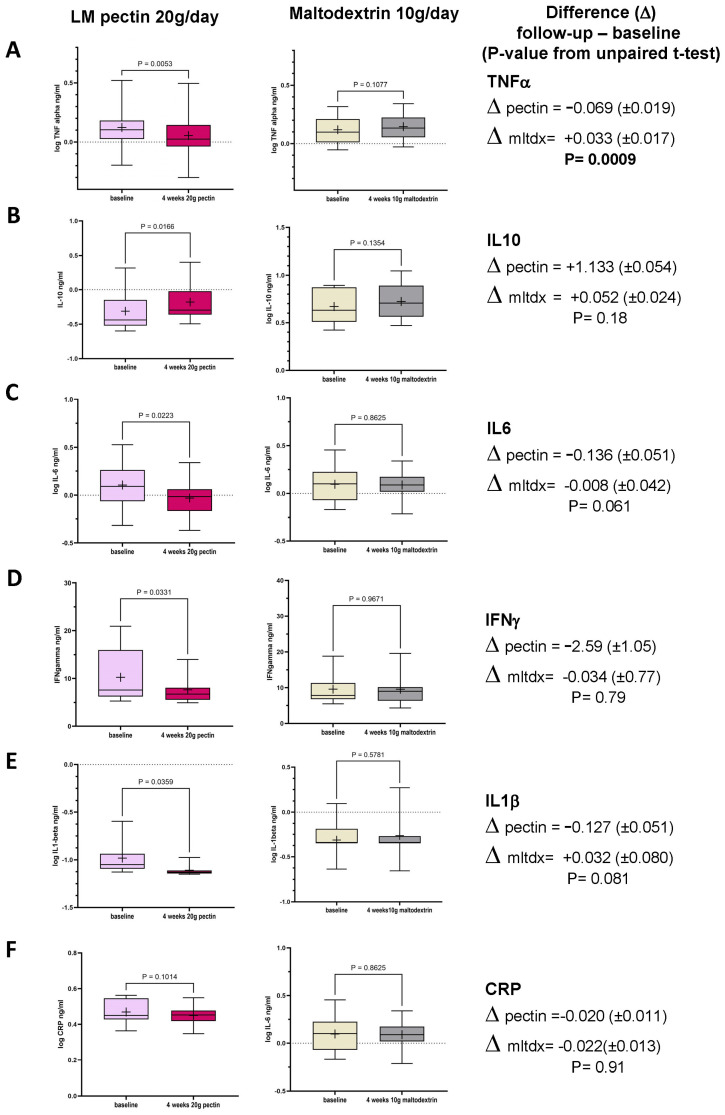
Box plots in circulating markers seen in the treatment group (n = 14; LM pectin 20 g/d) and the placebo group (n = 15, maltodextrin 10 g/d) for 4 weeks between baseline and follow-up for the six circulating molecules (**A**) TNF-alpha, (**B**) IL-10, (**C**) IL-6, (**D**) IFN-gamma, (**E**) IL-1beta and (**F**) hsCRP. Each box plot shows the mean (indicated by + in the plot), median (middle line), interquartile range (box) and 95% confidence intervals (whiskers) for the serum marker. *p*-values on each pair of box plots correspond to paired *t*-tests between baseline and follow-up. The right-most column shows the mean difference between baseline and follow-up (±standard error). The *p*-value is derived from the unpaired *t*-test between the LM pectin and placebo arms.

In the first interventional cohort, we observed changes in all the inflammatory markers tested in serum between baseline and follow-up (Figure 2A–E), as a result of consuming 20 g/d pectin for 4 weeks, with the exception of hsCRP (Figure 2F). None of the markers changed significantly in the placebo arm (Figure 2). When the change in markers was compared between both arms, only TNF-alpha (Figure 2A, *p* < 0.0009) showed a statistically significant difference between pectin and placebo, though the difference between pectin and placebo for both IL-1 beta and IL-6 achieved *p* < 0.10.

**Figure 3:**
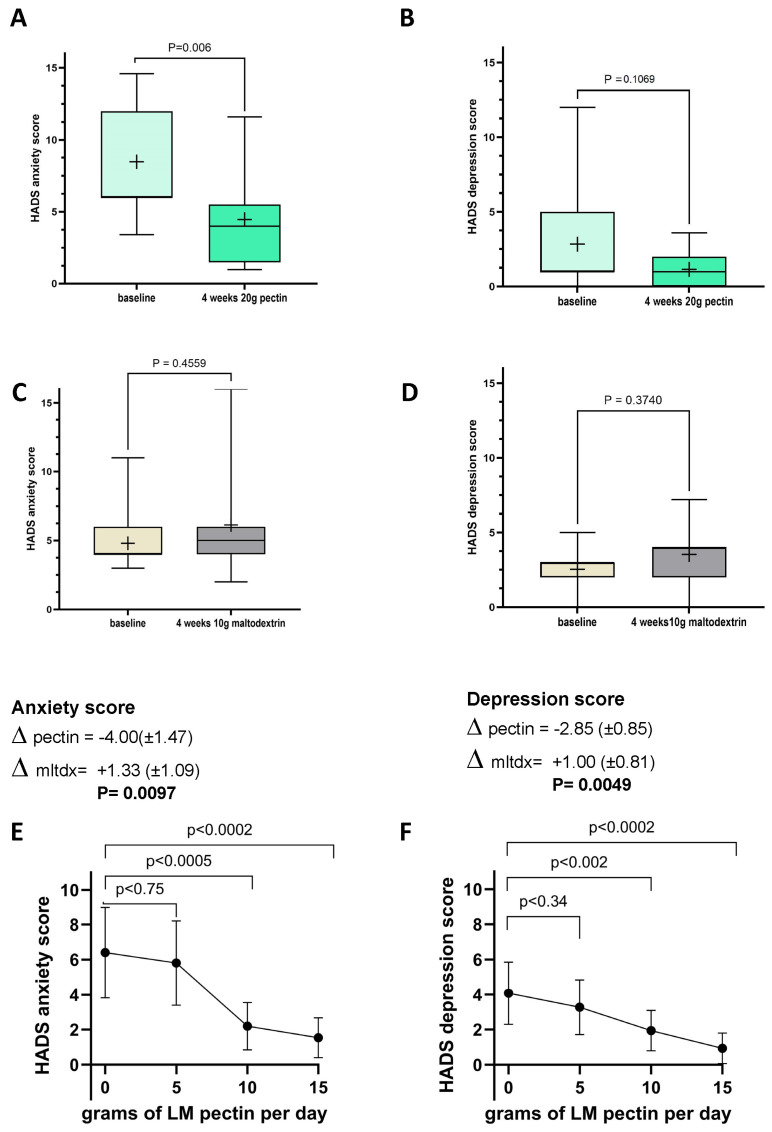
Changes in Hospital Anxiety and Depression Scale (HADS) scores between baseline and follow-up in intervention 1 with 20 g of LM pectin for (**A**) anxiety and (**B**) depression, with 10 g of maltodextrin for (**C**) anxiety scores and (**D**) depression scores and with increasing doses of LM pectin in intervention 2 for (**E**) anxiety and (**F**) depression. Each box plot shows the mean (indicated by + in the plot), median (middle line), interquartile range (box) and 95% confidence intervals (whiskers) for the serum marker. *p*-values on each pair of box plots correspond to paired *t*-tests between baseline and follow-up. The graphs for anxiety scores (**E**) and depression scores (**F**) show the mean (indicated by a dot) for each dose of LM pectin and 95% confidence intervals (error bars). *p*-values correspond to paired *t*-tests between increasing doses of LM pectin compared to baseline (i.e., 0 g).

In this first intervention, we also observed a significant reduction in anxiety scores (Figure 3A) in the pectin group, between baseline and follow-up, though the numerical differences in depression scores seen after the intervention in this group did not achieve statistical significance (Figure 3B). No changes to these variables were found in the control group (Figure 3C,D). The difference in HADS anxiety and depression scores observed between the baseline and follow-up and between the pectin and placebo arms was statistically significant for both anxiety and depression (*p* = 0.0097 and *p* = 0.0049, respectively).

We also tested whether there were changes in serum levels of TLR-2 and of zonulin, a marker of gut inflammation, and found no significant change in TLR-2 in either the treatment or pectin arm and a nominally significant drop in zonulin in the pectin arm (Table 2). The difference between both arms was not statistically significant for either marker.

Since pectin is a form of dietary fibre, we tested whether these effects were linked to increased production of short-chain fatty acids (SCFAs). SCFAs are carboxylic acids mainly produced by colonic bacteria through the fermentation of resistant polysaccharides which escape digestion and absorption [21]. SCFAs have anti-inflammatory effects via a number of mechanisms [22]. We therefore assessed fasting serum levels of SCFAs before and after pectin supplementation. We found, however, that there were no changes in SCFA serum concentrations with either pectin supplementation or in the placebo arm (Table 2).

Given the strong effects of 20 g per day of pectin supplement found on inflammatory markers and anxiety, we then decided to test the dose responsiveness of these effects in a separate cohort of healthy individuals. Due to a different recruitment strategy targeting young people, the second intervention cohort was younger (mean age = 36.0 years) and had a lower BMI (mean BMI = 23.5 kg/m^2^) than the initial intervention (Table 1). No significant changes were seen on the prevalence or intensity of gastrointestinal symptoms from baseline to any of the increasing pectin dosages (Table S1).

**Figure 4:**
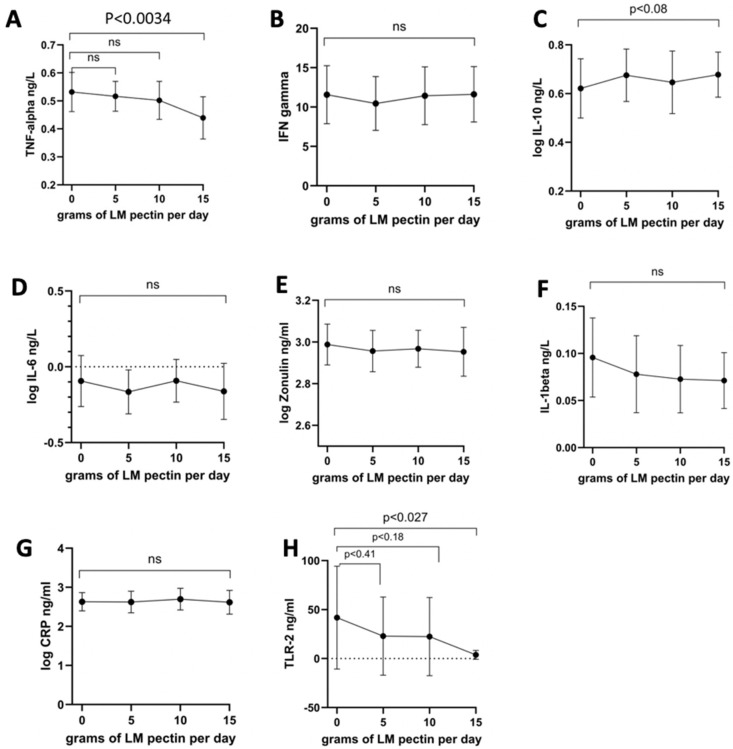
Changes in circulating markers seen with increasing doses of LM pectin in the second intervention study for the following circulating molecules: (**A**) TNF-alpha, (**B**) IFN-gamma, (**C**) IL-10, (**D**) IL-6, (**E**) zonulin, (**F**) IL-1beta, (**G**) hsCRP and (**H**) TLR-2. The graphs show the mean (indicated by a dot) for each dose of LM pectin and 95% confidence intervals (error bars). *p*-values correspond to paired *t*-tests between increasing doses of LM pectin compared to baseline (i.e., 0 g). ‘ns’ refers to not-significant.

In the second intervention cohort, the only proinflammatory cytokine to significantly decrease was TNF-alpha after 2 weeks of ingesting 15 g/d of LM pectin (Figure 4A). In fact, we found that most proinflammatory cytokine levels remained constant at 5, 10 and 15 g supplementation, although with some slight non-significant drops, e.g., for IL-1beta (Figure 4F). A not statistically significant (*p* < 0.08) trend towards higher levels of the anti-inflammatory cytokine IL-10 were also observed with 15 g of pectin (Figure 4C). The other nominally significant effect that we observed was a decrease in serum levels of TLR-2 with the 15 g dose (Figure 4H). On the other hand, we observed significant dose-dependent effects on both anxiety and depression scores with pectin supplementation. Significant drops in both scores were seen with 10 g and 15 g doses of LM pectin. Small drops were also found with 5 g but did not achieve statistical significance (Figure 3E,F).

## 4. Discussion

In this study we report, for the first time and to the best of our knowledge, the potential anti-inflammatory and anxiety-modulating effects of dietary supplementation with LM pectin in humans. We report widespread anti-inflammatory effects on a vast range of cytokines with a large daily dose (20 g), but only one of the cytokines, TNF-alpha, dropped significantly more than in the placebo arm and was also significantly decreased with a 15 g dose. None of the inflammatory markers were significantly lower with the 10 g dose compared to the baseline, indicating that the anti-inflammatory effects are dose-dependent and require fairly large doses of pectin.

We observed no difference in SCFA serum levels, although it is possible that SCFA production derived from pectin fermentation by gut microbes might have increased. Given that stool and serum levels have been shown to be very poorly correlated with each other [22] and that we did not measure stool levels of SCFAs, it is nonetheless possible that there may be changes in SCFA production. Importantly, we cannot exclude that the effects reported might still be mediated by changes in the gut microbiome composition resulting from pectin supplementation, which may not be related to changes in SCFA producers. For example, supplementation with high doses of high-methoxyl pectin (but not with pectin DM < 40) in murine inflammatory models results in significant increases in the genera *Prevotella* [23]. Although this genus consumes dietary fibre, it can generate mostly propionate, which represents <20% of SCFAs and is known to be quickly and efficiently consumed by hepatic metabolism, being a significant contributor to liver gluconeogenesis [24]; hence, changes in serum levels of this SCFA might be difficult to detect.

Regardless of changes to the gut microbiome (not measured here), the anti-inflammatory mechanism is likely to be caused at least in part by the TLR-2 binding mechanism previously reported in animal models [5]. Although the drop in TLR-2 seen in the 20 g cohort was not statistically significant, it was significant in the 15 g group. The ELISA assay measures accessibility of the TLR-2 molecule by monoclonal antibodies and not TLR-2 activity (which would have required obtaining peripheral blood mononuclear cells (PBMCs) and carrying a functional assay stimulating the PBMCs with known TLR-2 ligands such as bacterial lipopeptides). Therefore, the effects reported here are consistent with TLR-2 antagonism by LM pectin, although they do not necessarily prove that this is the mechanism taking place in humans and further functional assays would be required to demonstrate this.

We also report significant decreases in anxiety and depression scores within the normal (not clinical) range of these scales. These effects were clearly dose-dependent and were significant with doses as low as 10 g. Importantly, none of these effects were seen in the control group. Although a number of studies have hinted at possible effects of pectin on anxiety in animal models, to the best of our knowledge this is the first study showing significant drops in anxiety scores in humans. Interestingly, the drops in anxiety and depression are observed for the 10 g dose, whereas significant drops in inflammation are only found with doses of 15 g or higher. It is thus possible that additional mechanisms not directly linked to pectin’s anti-inflammatory effects, e.g., effects on the gut microbiome unaccounted for by SCFAs, may be responsible for the observed anxiety-reducing properties of pectin supplementation.

Interestingly, a recent study analysing metagenomic features linked to depression in inflammatory bowel disease (IBD) patients found that genes involved in pectin metabolism were strongly associated with lower HADS depression scores [25]. This suggests that specific microbial pathways which are not directly linked to SCFA production may also be involved in the anxiety-relieving effects of pectin [26]. This is in line with what is known of how gut dysbiosis may contribute to mental health issues like anxiety through mechanisms involving inflammation, neurotransmitter dysregulation [27], HPA axis dysfunction [28], gut–brain communication [29] and altered tryptophan metabolism [27].

The effect of pectin on anxiety scores in humans opens a number of potential clinical applications. Given the strong safety profile of pectin, nutritional supplementation with this natural polysaccharide represents a safe and cost-effective non-pharmacological approach for enhancing the long-term mental well-being of individuals grappling with anxiety. However, given that LM pectin’s effects were tested in healthy volunteers, these findings cannot be extrapolated to clinical populations and future studies are needed to assess the role of pectin supplementation to assess whether the anxiety-reducing and anti-inflammatory effects are also observed in patient groups.

We used maltodextrin, a frequently used placebo in dietary intervention studies due to it being a tasteless, odourless, easily dissolvable polysaccharide [30] with minimal physiological effects at moderate doses [31], thus making it a suitable comparator to the active intervention (i.e., pectin) arm.

Our study has several important strengths. It included a control group in which none of the effects of pectin were found. It also tested the effect of pectin in two independent cohorts and in a dose–response manner, which strengthens the validity of the conclusions. It used clinically validated instruments such as the HADS and the GSRS questionnaires to assess clinical outcomes and it measured key inflammatory markers in fasting serum samples.

We also note several study limitations. The sample size, although sufficient for the effect sizes found, is still relatively modest and it is possible that larger effect sizes might have detected some of the inflammatory changes seen at the 15 and 10 g doses as statistically significant. It is well known that small sample sizes can result in both false positive and false negative errors. Given that the effects on anxiety and TNF-alpha levels were replicated in a second cohort at a lower dose, these effects are unlikely to be false positives. However, the lack of significant effects on some of the inflammatory markers or on SCFAs could be due to a lack of statistical power. Another important limitation is the lack of PBMCs for testing TLR-2 activity, which means that we cannot definitely ascertain a link between the observed anti-inflammatory effects and this mechanism. We also note the lack of gut microbiome composition data. Although our results indicate no changes in SCFA serum levels, that does not preclude that the gut microbiome might be mediating some of the effects seen. In fact, different effects of LM and HM pectin have been reported in animal models (see, e.g., [23]). Due to the large number of species that can change in response to different carbohydrate sources, a more comprehensive analysis with sufficient statistical power is needed to assess any microbiome-mediated mechanisms involved in the effects observed in humans resulting from LM pectin supplementation. Future research collecting PBMCs, stool samples and with larger sample sizes are needed to answer questions regarding the specific mechanisms involved.

Another potential limitation is that a large proportion of the volunteers are female, which may limit generalisability given that women are almost twice as likely to experience anxiety disorders compared to men [32]. More balanced groups or male-only studies would be needed to assess whether the effects on mood are also significant in men. Finally, supplementation lasted 4 weeks at 20 g or 6 weeks at increasing doses; hence, we cannot assess the effects of long-term supplementation with LM pectin. Therefore, although we did not see any effects of supplementation for two weeks with 5 g followed by two weeks with 10 g of LM pectin, that does not preclude that, e.g., 6 or 8-week supplementation with 10 g might have resulted in anti-inflammatory changes being detected. Larger and longer-term intervention studies are required to clarify the exact doses.

## 5. Conclusions

In conclusion, in this pilot study, we have shown that supplementation with LM pectin results in significant drops in TNF-α at 20 g and 15 g per day and in significant drops in anxiety and depression scores at 10 g, 15 g and 20 g per day in healthy humans. No effect on circulating SCFAs or CRP, but modest drops of other inflammatory markers, were seen at 20 g/day but not at lower doses. The anti-inflammatory effects are consistent with the known antagonising role of LM pectin on TLR2 but the effects on mood might also be influenced by gut microbial changes that were not measured in this pilot study. Although these results suggest promising potential for LM pectin supplementation to improve mood and reduce some markers of systemic inflammation, further research is needed to assess whether these effects are also observed in patient populations (e.g., for mental health or for any number of diseases with a strong component of systemic inflammation). In addition, future studies are needed to elucidate the precise mechanism of the effects of LM pectin on mood, to validate the efficient dose and minimum duration of supplementation and to assess whether these effects are seen in any patient populations.

## Figures and Tables

**Table 1:** Descriptive characteristics for interventional cohort 1 and 2.

**Intervention 1 (n = 29)**							
**Variable**	**Pectin Arm (n = 14)**		**Control Arm (n = 15)**		***p*-Value from *t*-Test**		
Age (yrs)	40.3 (±16.41)		43.2 (±11.4)		0.59		
Sex, F%	57%		66%		0.61		
BMI (kg/m2)	24.54 (±2.99)		25.18 (±2.06)		0.63		
**Intervention 2 (n = 15)**							
**Variable**	**Baseline (±SD)**	**Week 2 after 5 g/d (±SD)**	**Week 4 after 10 g/d (±SD)**	**Week 6 after 15 g/d (±SD)**	***p*-Value 0 vs. 5 g**	***p*-Value 0 vs. 10 g**	***p*-Value 0 vs. 15 g**
Age (yrs)	36.0 (±20.82)	-	-	-	-	-	-
Sex, F%	80%	-	-	-	-	-	-
BMI (kg/m2)	23.51 (±2.41)	23.53 (±2.55)	23.63 (±2.38)	23.84 (±2.49)	0.92	0.42	0.35

**Table 2:** Fasting serum TLR-2, zonulin and SCFA concentrations in the pectin and control arms for intervention cohort 1.

	Pectin Arm (n = 14)			Control Arm (n = 5)		
	Baseline (±SD)	Follow-Up (±SD)	*p* Value	Baseline (±SD)	Follow-Up (±SD)	*p* Value
TLR-2 (ng/mL)	24.13 (±67.73)	21.35 (±62.16)	0.323	27.06 (±71.70)	18.43 (±64.85)	0.625
Zonulin (ng/mL)	35.09 (±10.55)	29.19 (±10.62)	0.041	25.65 (±5.49)	23.31 (±7.84)	0.872
Acetic acid (ng/mL)	2556 (±1387)	3038 (±1948)	0.501	1857 (±304)	2070 (±666.9)	0.625
Butyric acid (ng/mL)	715.8 (±611)	582.1 (±528.3)	0.463	518.6 (±4.587)	101.1 (±19.18)	0.375
2-Methylbutyric acid (ng/mL)	119.5 (±59.07)	127.8 (±69.96)	0.569	115.2 (±36.39)	125.80 (±9.17)	0.625
Propionic acid (ng/mL)	290.5 (±69.48)	293.4 (±72.29)	0.727	303 (±35.52)	246 (±24.99)	0.250
Iso-butyric acid (ng/mL)	515.5 (±351)	464.4 (±338.2)	0.541	314 (±382.6)	789.5 (±52.86)	0.250
Iso-valeric acid (ng/mL)	99.82 (±31.01)	90.45 (±24.81)	0.273	84.10 (±23.56)	89.63 (±27.31)	0.875
Valeric acid (ng/mL)	27.39 (±7.77)	28.10 (±5.16)	0.357	32.35 (±9.16)	28.28 (±6.64)	0.500
Hexanoic acid (ng/mL)	82.93 (±34.74)	99.15 (±48.11)	0.951	71.20 (±34.22)	78.40 (±32.04)	0.875

## Data Availability

Data can be made readily available to bona fide researchers upon writing to the corresponding author.

## Supplementary Materials

The following supporting information can be downloaded at: https://www.mdpi.com/article/10.3390/nu16193326/s1, Table S1: Gastrointestinal Symptom Rating Scale (GSRS) scores in intervention 2.
