# Supplementary material for: Supplementation with Citrus Low-Methoxy Pectin Reduces Levels of Inflammation and Anxiety in Healthy Volunteers: A Pilot Controlled Dietary Intervention Study
Source: Nutrients. 2024 Sep 30;16(19):3326. doi: 10.3390/nu16193326 (PMC11479230; doi:10.3390/nu16193326)
Supplement: Supplementary file 1 [file nutrients-16-03326-s001.zip › nutrients-3202923-supplementary.pdf]

Supplementary Table S1: Gastrointestinal Symptom Rating Scale (GSRS) scores in intervention 2.

**Intervention 2**							
**variable**	**Baseline (±SD)**	**Week 2 after 5g/d (±SD)**	**Week 4 after 10g/d (±SD)**	**Week 6 after 15g/d (±SD)**	p-value 0 vs 5g	p-value 0 vs 10g	p-value 0 vs 15g
GSRS	2.60 (±2.68)	2.80 (±4.08)	3.93 (±4.90)	3.07 (±4.34)	*0.584*	*0.69*	*0.97*
